# Mindfulness-based cognitive therapy for depressed individuals improves suppression of irrelevant mental-sets

**DOI:** 10.1007/s00406-016-0746-x

**Published:** 2016-11-09

**Authors:** Jonathan Greenberg, Benjamin G. Shapero, David Mischoulon, Sara W. Lazar

**Affiliations:** 1 Department of Psychiatry, Massachusetts General Hospital, Harvard Medical School, 120 2nd Ave, Charlestown, MA 02129 USA; 2 Department of Psychiatry, Massachusetts General Hospital, Harvard Medical School, 1 Bowdoin Square, 6th Floor, Boston, MA 02114 USA

**Keywords:** Mindfulness-based cognitive therapy, Depression, Mental-set, Competitor rule suppression

## Abstract

An impaired ability to suppress currently irrelevant mental-sets is a key cognitive deficit in depression. Mindfulness-based cognitive therapy (MBCT) was specifically designed to help depressed individuals avoid getting caught in such irrelevant mental-sets. In the current study, a group assigned to MBCT plus treatment-as-usual (*n* = 22) exhibited significantly lower depression scores and greater improvements in irrelevant mental-set suppression compared to a wait-list plus treatment-as-usual (*n* = 18) group. Improvements in mental-set-suppression were associated with improvements in depression scores. Results provide the first evidence that MBCT can improve suppression of irrelevant mental-sets and that such improvements are associated with depressive alleviation.

**Electronic supplementary material:**

The online version of this article (doi:10.1007/s00406-016-0746-x) contains supplementary material, which is available to authorized users.

## Introduction

Depression is a leading cause of disability and one of the most common mental disorders [1]. It is characterized by impaired ability to suppress competing or currently irrelevant mental-sets [2–8], such as distracting ruminative thoughts. Impairments in mental-set suppression have been found to predict onset and recurrence of depression, correlate with depressive rumination [9, 10], and mediate symptom severity [4, 11]. However, little is known about whether or how suppression impairments can be improved and if this would affect depressive symptomatology. This study aims to determine whether mindfulness training can improve mental-set suppression and whether such improvement is associated with depressive alleviation.

Mindfulness-based cognitive therapy (MBCT) specifically targets avoiding getting caught in ruminative mental-sets [12–14] and can prevent depressive relapse [13, 15]. While mindfulness training can improve cognitive functioning [16] including mental-set suppression [17–19] primarily among healthy adults, cognition improvements among depressed individuals remain largely unexplored.

The current study focuses on *Competitor Rule Suppression* CRS [20] as a measure of mental-set suppression. CRS refers to suppression of mental-sets which implicate a response that competes with the correct or currently relevant response (for example, suppressing self-critical thoughts of giving up rather than staying focused on a difficult task). CRS specifically counters “troublemaking” irrelevant mental-sets by tagging them in episodic memory as “to-be-suppressed” [21], thus facilitating adherence to current task demands. CRS is measured within a task-switching paradigm [22, 23], in which the context and task requirements are in constant flux. This design permits examination of dynamic fine-tuning of suppression processes [20, 24, 25], rather than more crude and consistent suppression of a single process or stimulus seen in other suppression measures such as the Stroop [20, 26]. We hypothesized that MBCT will improve CRS and that such improvements will be linked to depressive symptom reduction.

## Methods

### Participants

**Fig. 1:**
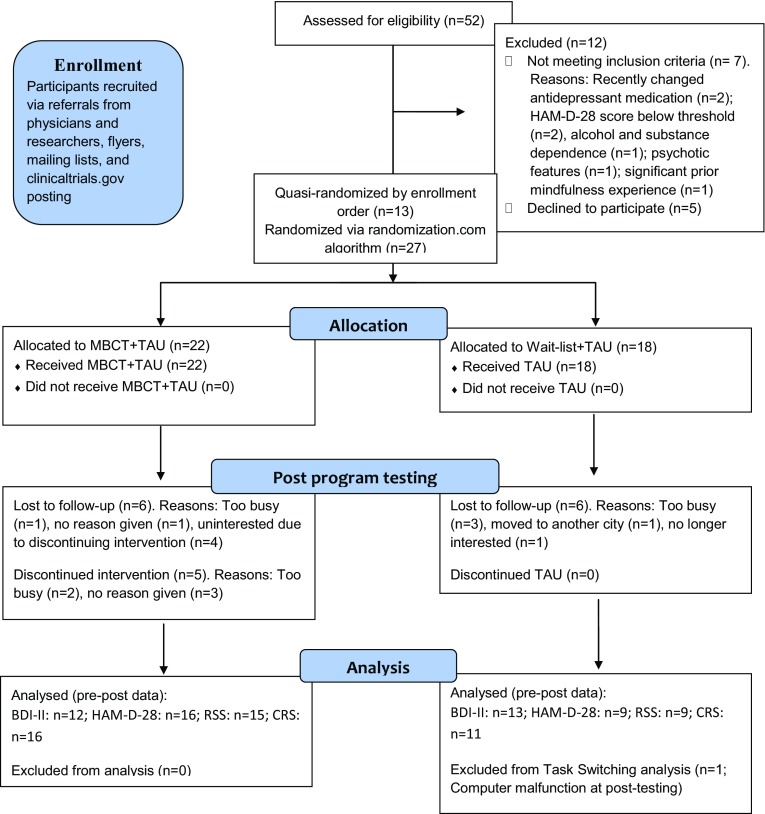
Participant flow

Fifty-two participants with mild to severe depression were recruited (Fig. 1). Inclusion criteria included a score of ≥11 on the 28-item Hamilton Depression Scale (HAM-D-28; [27]), dysphoria or low mood for at least two months prior, no history of substance dependence or abuse, psychotic features, or suicidal attempts in the past six months, and no prior experience with systematic mindfulness programs. Antidepressants were allowed only if doses were stable for ≥6 weeks. Forty participants were assigned to MBCT + treatment-as-usual (TAU) or wait-list + TAU. The first 13 participants were quasi-randomized and assigned in order of enrollment. Following an increase in recruitment, the next 27 participants were randomized after completing baseline testing. Groups were statistically equivalent in age (*t*(38) = 0.69, ns) and gender, meeting MDD criteria, and having a comorbid anxiety condition (minimal *p* = 0.44; Fisher’s exact test; Table 1). The study was approved by the Massachusetts General Hospital’s Institutional Review Board and registered at clinicaltrials.gov (NCT02457936).

**Table 1:** Baseline demographic and clinical characteristics of participants (all baseline group differences are nonsignificant; minimal *p* = 0.49)

	MBCT + TAU	Wait-list + TAU
Gender	59% Women	66% Women
*Age*		
Mean	39.77	36.89
SD	10.6	15.83
*BDI-II score*		
Mean	23.51	22.18
SD	7.09	9.39
*HAM-D-28 score*		
Mean	22.80	24.00
SD	8.62	6.34
Meeting major depressive disorder criteria (%)	86	72
Comorbid anxiety disorder (%)	54	44

### Mindfulness-based cognitive therapy program

The 8-week MBCT program was led by two MBCT teachers with 8–13 years experience of teaching mindfulness-based group programs and blind to the study’s hypotheses. The program followed the guidelines of Segal, Williams, and Teasdale [12].

### Measures

#### Clinical measures

The *Mini International Neuropsychiatric Interview* [28] version 5.0.0 and the HAM-D-28; [27, 29] were administered by trained and certified psychiatrists and psychologists from Mass. General Hospital to assess inclusion criteria. The Beck Depression Inventory-II (BDI-II [30]) was administered 0–2 weeks prior to the MBCT program and every 2–3 weeks during the program to measure changes in depressive symptoms. The Rumination Response Scale was also administered. These data are detailed in the electronic supplementary material.

#### Competitor rule suppression

**Fig. 2:**
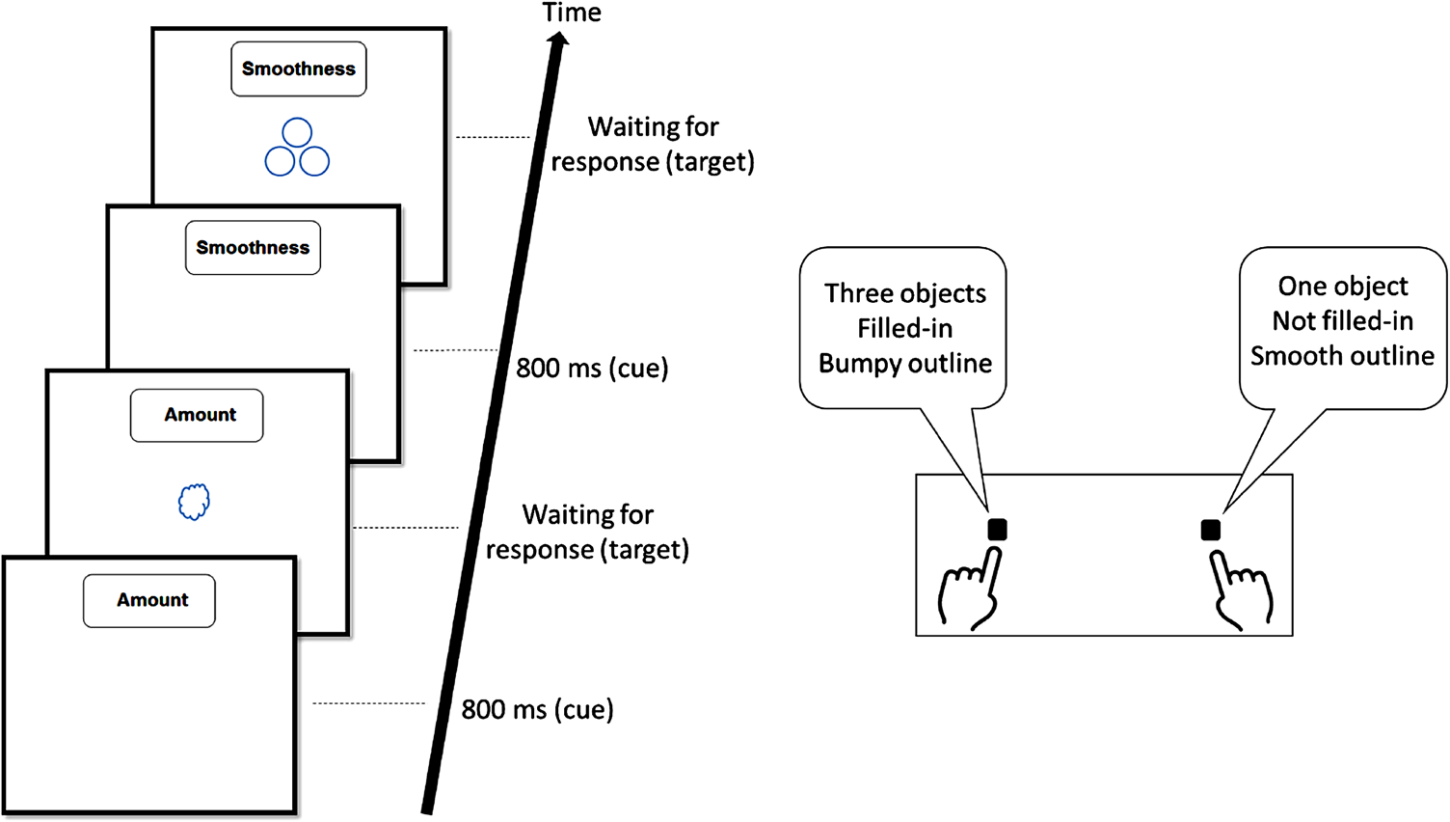
Illustration of events in the task-switching paradigm. In the first presented trial, the relevant rule is “Amount” and correct answer is the *right key*. “Smoothness” in this trial is a conflicting rule as it indicates the *left key* as the correct response. CRS is evident by hampered performance (indicating rule suppression) when on the following trial the previously conflicting rule (“Smoothness”) becomes the relevant rule, as in the illustrated example

CRS was assessed via a task-switching paradigm based on [31] (Fig. 2). Briefly, a text-cue indicated which of three tasks was to be performed (“Amount” to indicate whether one or three objects are presented, “Filled-In” to indicate whether the object is filled with color or not, or “Smoothness” to indicate whether the object’s outline is smooth or bumpy). Tasks were presented in random order, with no repetition on two consecutive trials in order to maximize the number of CRS trials [19, 20]. Participants completed 20 practice trials, followed by three experimental blocks of 120 trials each. CRS was calculated by subtracting reaction time (RT) and error rates in trials in which the current rule was not the competing rule in the previous trial (non-CRS trials) from trials in which the current rule was the competing rule in the previous trial (CRS trials; [19–21, 32]; Fig. 2). RT analyses were performed on trials in which the current and last responses were correct. RT of over 3500 ms (ms) was excluded [19]. Information regarding *Backward Inhibition,* an additional measure assessed with the same task, is detailed in the electronic supplementary material.

### Procedure

Participants completed a phone screen, then signed the consent form, and underwent the MINI and the clinician-rated HAM-D-28 to assess eligibility. They then completed the CRS task, the BDI-II, and other measures outside the scope of this report. Testing procedures were repeated for all participants 0–3 weeks after the MBCT program. Clinical assessors were blind to group allocation.

## Results

### Depression scores

#### BDI-II

**Fig. 3:**
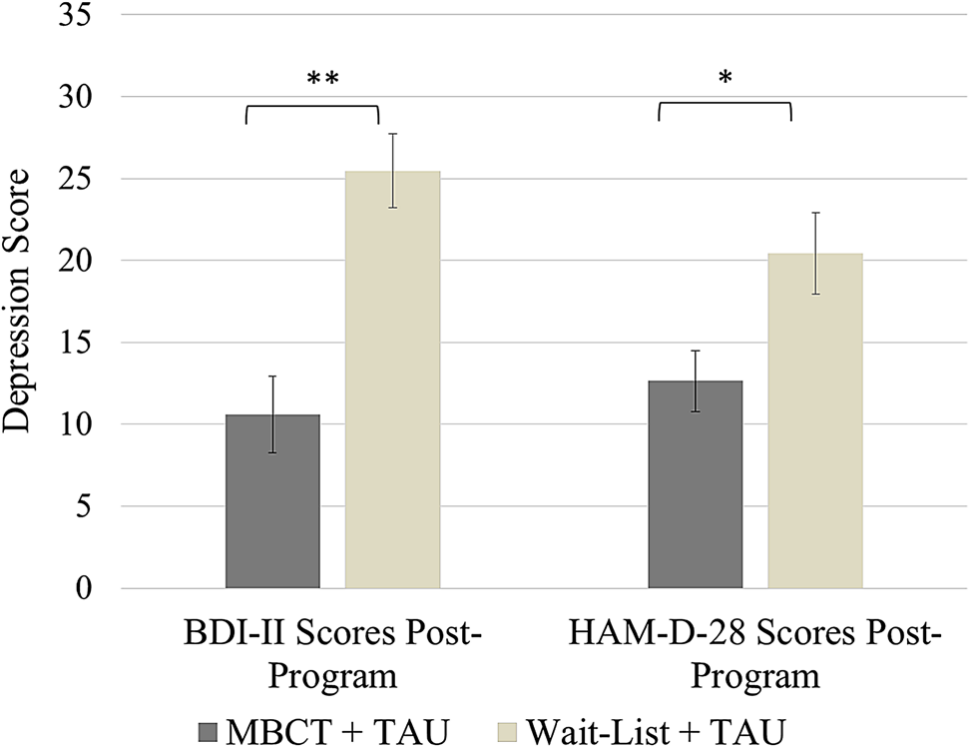
ANCOVAs of BDI-II and HAM-D-28 scores post-program with baseline scores as covariates; **F(1,22) = 22.51, *p* < 0.001; *F(1,22) = 4.77, *p* < 0.05

Groups had statistically equivalent BDI-II scores at baseline, both when examining all participants (*t*(36) = 0.50, ns) and participants with post-program data, (*t*(23) = 0.16, ns; Table 1). A one-way analysis of covariance (ANCOVA) with group as the independent variable conducted on post-program BDI-II scores while controlling for baseline BDI-II scores revealed a highly significant effect for group, with MBCT + TAU (*M* = 10.60, *SD* = 8.12) exhibiting lower BDI-II scores post-program than controls (*M* = 25.46, *SD* = 14.91; *F*(1,22) = 22.51, *p* < 0.001, *η*_*p*_^2^ = 0.505, 90% CI (0.23, 0.65); Fig. 3). BDI-II scores from week 6 were used for four participants who discontinued before post-testing. This effect remained highly significant after excluding these participants *F*(1,18) = 29.14, *p* < 0.001, *η*_*p*_^2^ = 0.618, 90% CI (0.33, 0.74).

#### HAM-D-28

Groups had statistically equivalent baseline HAM-D-28 scores, both when examining all participants *t*(35) = 0.47, ns (Table 1) and when examining only participants with post-program data (*t*(23) = 0.71, ns). ANCOVA conducted on post-program HAM-D-28 scores while controlling for baseline scores revealed a significant effect for group, with MBCT + TAU (*M* = 12.63, *SD* = 8.76) exhibiting lower HAM-D-28 scores post-program than wait-list + TAU (*M* = 20.44, *SD* = 6.35), *F*(1,22) = 4.77, *p* < 0.05, *η*_*p*_^2^ = 0.178, 90% CI (0.005, 0.39; Fig. 3).

#### CRS

**Fig. 4:**
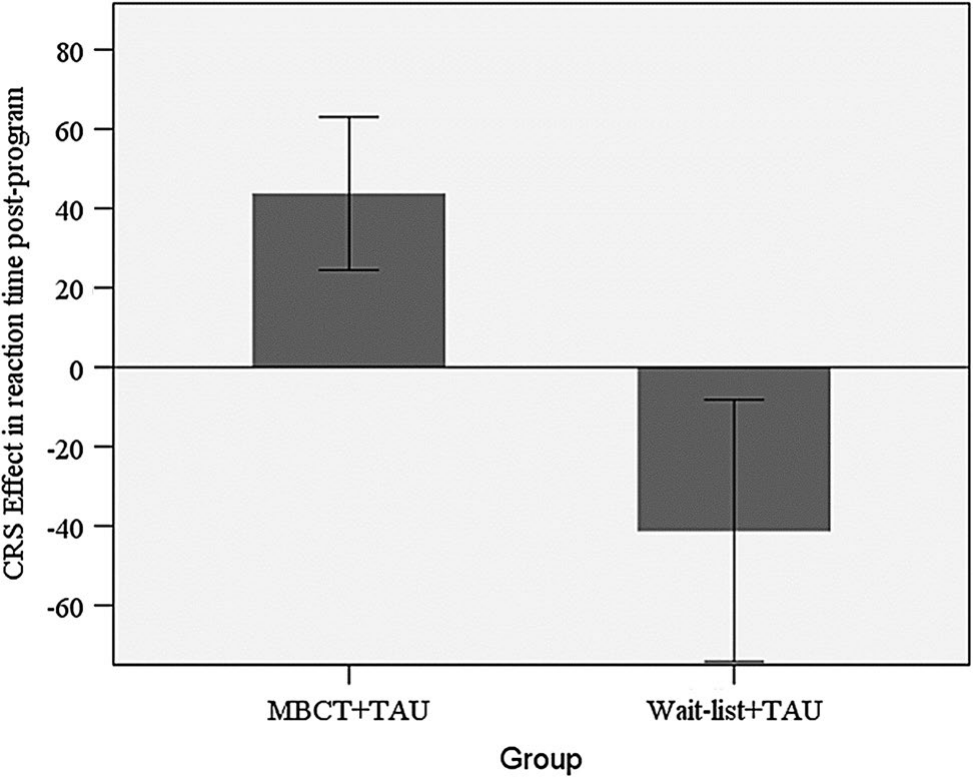
ANCOVA of CRS in RT post-program with baseline scores as a covariate; *F(1,24) = 6.13, *p* = 0.02

Groups were equivalent at baseline in reaction time (RT; *t*(38) = 0.36, ns) and error rates (*t*(38) = 1.00, ns) when including all participants and when including only participants with post-program data (maximal *t*(25) 1.38, ns). An ANCOVA with group as the independent variable conducted on post-program RT while controlling for baseline RT revealed a significant effect for group, with the MBCT + TAU group (*M* = 44 ms, *SD* = 77 ms) exhibiting an increased CRS effect compared to the control group (*M* = −41 ms, *SD* = 109 ms; *F*(1,24) = 6.13, *p* = 0.02, *η*_*p*_^2^ = 0.20, 90% CI (0.02, 0.40); Fig. 4). A similar ANCOVA conducted on post-program error rates while controlling for baseline error rates did not reach significance (*F*(1,24) = 1.57, ns).

### The relationship between CRS and depressive symptoms

Multiple regression was used to predict change in BDI-II based on change in CRS error rates and RT. A significant regression model was found (*F*(1,19) = 6.91, *p* = 0.02) with change in error rates associated with change in depressive symptoms. The multiple correlation coefficient was 0.52, indicating that overall improvement in irrelevant mental-set suppression explained 26.7% of the variance in BDI-II scores (*β* = 0.52*, p* = 0.02*; R* ^2^ = 0.267). A similar regression was calculated with group as an additional factor. A significant model was found (*F*(2,18) = 19.20, *p* < 0.001), with a multiple correlation coefficient of 0.82 (*R* ^2^ = 0.64). Group (*β* = 0.68*, p* < 0.001) was a significant factor and error rates just attained significance (*β* = 0.30*, p* = 0.05).

## Discussion

This study found significantly lower depression scores and significantly higher CRS following MBCT compared to wait-list + TAU. Moreover, improvements in CRS were significantly associated with improvements in BDI-II scores. These results constitute the first empirical evidence indicating MBCT can help improve mental-set suppression, as well as the first evidence linking such improvements to depressive alleviation.

CRS has been labeled “smart inhibition” [33] since it measures the ability to detect and target only conflicting and “troublemaking” mental-sets. CRS operates on a higher order and abstract level than most inhibition measures in the sense that it does not merely involve suppression a competing *response* but of a competing *rule,* regardless of the specific response [20, 21]. This type of finely tuned suppression mechanism fits particularly well with the mindfulness training practiced in MBCT, which focuses on avoiding getting caught in depression-related thoughts that often conflict with one’s ability to focus on current task demands. Our findings suggest that reductions in depressive symptoms are associated with such specialized and specific suppression. These benefits following improved specific suppression contrast with broad and more general thought suppression among depressed individuals, which may prolong or worsen depressive symptoms [34, 35].

The primary limitation of this study was the small sample. Possibly some null effects would have reached significance in a larger sample. The small sample is somewhat less problematic with regard to the significant differences found in this study, since the more probable statistical error in this case is of type I. Second, the use of a wait-list rather than an active control program limits us to only attribute findings to the MBCT program as a whole, rather than to mindfulness training specifically. Finally, although group assignment for most participants was random, the first few participants were quasi-randomized by enrollment order.

Although the current results should be confirmed in larger samples, they provide preliminary evidence that MBCT can improve mental-set suppression, a key cognitive deficit in depression. Furthermore, these findings demonstrate that these improvements in are associated with improvements in depressive symptoms.

## Electronic supplementary material

Below is the link to the electronic supplementary material.

Supplementary material 1 (PDF 214 kb)
