# Supplementary material for: Mindfulness-based cognitive therapy for depressed individuals improves suppression of irrelevant mental-sets
Source: Eur Arch Psychiatry Clin Neurosci. 2016 Nov 9;267(3):277–82. doi: 10.1007/s00406-016-0746-x (PMC5357295; doi:10.1007/s00406-016-0746-x)
Supplement: Supplementary file 1 — Supplementary material 1 (PDF 214 kb) [file 406_2016_746_MOESM1_ESM.pdf]

Mindfulness-Based Cognitive Therapy for Depressed Individuals Improves Suppression of Irrelevant Mental-Sets

Electronic Supplementary Material

European Archives of Psychiatry and Clinical Neuroscience

Jonathan Greenberg, Benjamin G. Shapero, David Mischoulon, and Sara W. Lazar, Department of Psychiatry, Massachusetts General Hospital & Harvard Medical School

Correspondence concerning this article should be addressed to Jonathan Greenberg; jgreenberg5@mgh.harvard.edu; jongreen80@gmail.com; 120 2nd Ave, Charlestown MA, 02129.

Below we report results from two additional measures collected in the current study - Backward Inhibition[1] and the Rumination Response Scale (RRS[2]).

Backward Inhibition is an index which can be extracted from the task-switching paradigm used in the current study, and reflects residual inhibition from recent but currently irrelevant mental sets[1, 3]. Switching from one task to another requires inhibition of the current task set [1, 4]. This inhibition can be quantified by impaired performance when returning to a recently inhibited task (e.g. A-B-**A** task sequence) compared to performance on a task which has not been recently been inhibited (e.g. C-B-**A** task sequence). Backward Inhibition is therefore calculated by subtracting reaction time (RT) and error rates in trials in which the current task was recently inhibited from trials in which the current task was not recently inhibited.

Groups were equivalent at baseline in Backward Inhibition RT $t(38)=1.85$, ns and error rates $t(38)=1.17$, ns when including all participants, as well as when including only participants with post-program data (RT $t(25)=1.22$, ns and error rates $t(25)=1.83$, ns). A one way ANCOVA on post-program Backward Inhibition RT with Group as the independent variable while controlling for baseline Backward Inhibition RT revealed no significant group differences post-program $F(1,24)=1.42$, ns. A similar ANCOVA revealed no group differences in error rates $F(1,24)=0.82$, ns.

To assess the relationship between changes in Backward Inhibition and changes in depressive symptoms, a multiple regression was calculated to predict change in the BDI-II, based on change in Backward Inhibition RT and error rates. No significant model was found (maximal $F(1,20)=1.00$, ns), indicating that change in Backward Inhibition does not within itself associate with change in BDI-II scores. A similar multiple regression with both CRS and Backward Inhibition RT and error rates as independent variables yielded a significant regression model

F(2,19)=8.47, *p*<0.01 with change in CRS and Backward Inhibition error rates as significant factors associated with change in depressive symptoms. The multiple correlation coefficient was 0.69, indicating that overall improvement in irrelevant mental set suppression explained 47.1% of the variance in BDI-II scores (R^2 =0.471). Increases in CRS significantly associated with improvements in BDI-II scores (β = 0.70, *p*<0.01) while increases in Backward Inhibition paradoxically showed an opposite effect, being associated with increases in BDI-II scores (β = -0.49, *p*=0.01). Similar results were obtained when excluding the four participants whose BDI-II scores were collected prior to program conclusion (*R*=0.69, R^2 =0.47, CRS β = 0.71, *p*=.001, Backward Inhibition β = -0.49, *p*=0.02).

Rumination Response Scale (RRS). The *RRS* is a 22 item questionnaire in which participants rate the degree of to which they tend to ruminate in response to depressive symptoms**.** Groups had equivalent rumination scores at baseline, both when examining all participants, *M*=58.90, *SD*=12.07 for the MBCT+TAU group; *M*=61.19, *SD*=10.91 for wait-list+TAU; *t*(36)=0.60, ns, and when examining baseline data only from participants with valid post-program data *M*=56.80, *SD*=11.18 for the MBCT+TAU group; *M*=60.50, *SD*=9.38 for wait-list+TAU; *t*(23)=0.86, ns. An ANCOVA with Group as the independent variable was conducted on post-program RRS scores while controlling for baseline RRS scores. The MBCT+TAU group (*M*=50.23, *SD*=13.39) did not significantly differ from the control group *M*=56.30, *SD*=7.57; *F*(1,22)=0.85, ns in rumination scores at post-testing. However, while rumination scores of the control group did not significantly change following treatment as usual (*F*(1,23)=2.01, ns), rumination scores of the MBCT+TAU were significantly reduced following MBCT (*F*(1,23)=7.31, *p*=0.01).

Baseline rumination scores significantly and positively correlated with baseline BDI-II scores (r=0.38, p<0.05). After the MBCT program, rumination scores showed a positive but non-significant trend of correlating with BDI-II scores (r=0.33, p=0.12). Changes in rumination from baseline to post-program did not significantly correlate with changes in BDI-II (r=0.05, ns).

Based on previous cross-sectional studies linking impairments in Backward Inhibition to rumination [5, 6], a regression model was calculated to assess the relationship between changes rumination scores and change in RT and error rates of Backward Inhibition and CRS. A significant model was found with change in Backward Inhibition error rates as a predictor of change in rumination $F(1,22)=4.73$, p<0.05. The multiple correlation coefficient was 0.42, indicating that changes in Backward Inhibition error rates explained 17.7% of the variance in rumination scores. Increases in Backward Inhibition was associated with reduction in rumination scores ($\beta = 0.42$, p<.05).

Discussion

Results from the measures of Backward Inhibition and the Rumination Response Scale indicate that MBCT+TAU did not improve Backward Inhibition or rumination scores compared to wailist+TAU. Additionally, while Backward Inhibition was associated with reduced rumination scores, it was paradoxically also associated with increased depressive symptoms when considered along with CRS. Given the current and previous [5, 6] findings linking impairments in Backward Inhibition to increased rumination, the correlation found between increased Backward Inhibition and elevated depressive symptoms was unexpected.

One possible explanation for this relates to the differential degree of specificity between CRS and Backward Inhibition. Backward Inhibition, in contrast to CRS, operates by broadly suppressing *any* recent mental set rather than just conflicting mental sets. The ability to broadly suppress recent mental sets reflected by Backward Inhibition may be helpful in order to initially "break away" from the repetitive thought patterns and therefore reduce rumination [5, 6]. However, several studies have demonstrated that other broad-based inhibition methods may result in contradictory and adverse effects, including prolonging or worsening of depressive symptoms[7–9]. This is particularly pronounced in stressful or cognitively demanding situations, partially due to a *post-suppression rebound* in which such thought suppression paradoxically produces a preoccupation with the suppressed thought when cognitive resources are limited. It is possible that a similar process is related to the association between backward inhibition and depressive symptoms found in the current study. This, however, is only a tentative post-hoc explanation which requires further examination. The differential relationship of Backward Inhibition with depressive symptoms and rumination is consistent with the previously expressed notion that although rumination is a common risk factor in depression [10], the two represent separate constructs [11] and may change differentially over the course of treatment.

References

1. Mayr U, Keele SW (2000) Changing internal constraints on action: the role of backward inhibition. J Exp Psychol Gen 129:4–26. doi: 10.1037/0096-3445.129.1.4

2. Nolen-Hoeksema S, Morrow J (1991) A prospective study of depression and posttraumatic stress symptoms after a natural disaster: the 1989 Loma Prieta Earthquake. J

Pers Soc Psychol 61:115–121. doi: 10.1037/0022-3514.61.1.115

3. Meiran N, Hsieh S, Dimov E (2010) Resolving task rule incongruence during task switching by competitor rule suppression. J Exp Psychol Learn Mem Cogn 36:992–1002. doi: 10.1037/a0019761

4. Koch I, Gade M, Schuch S, Philipp AM (2010) The role of inhibition in task switching: a review. Psychon Bull Rev 17:1–14. doi: 10.3758/PBR.17.1.1

5. Whitmer AJ, Banich MT (2007) Inhibition versus switching deficits in different forms of rumination. Psychol Sci 18:546–53. doi: 10.1111/j.1467-9280.2007.01936.x

6. Whitmer AJ, Gotlib IH (2012) Switching and backward inhibition in major depressive disorder: The role of rumination. J Abnorm Psychol 121:570–578. doi: 10.1037/a0027474

7. Wenzlaff RM, Wegner DM (2000) Thought Suppression. Depression 59–91.

8. Wenzlaff RM, Bates DE (1998) Unmasking a cognitive vulnerability to depression: how lapses in mental control reveal depressive thinking. J Pers Soc Psychol 75:1559–1571. doi: 10.1037/0022-3514.75.6.1559

9. Wenzlaff RM, Luxton DD (2003) The role of thought suppression in depressive rumination. Cognit Ther Res 27:293–308. doi: 10.1023/A:1023966400540

10. Nolen-Hoeksema S (1991) Responses to depression and their effects on the duration of depressive episodes. J Abnorm Psychol 100:569–582.

11. Smith JM, Alloy LB (2009) A roadmap to rumination: A review of the definition, assessment, and conceptualization of this multifaceted construct. Clin Psychol Rev 29:116–128. doi: 10.1016/j.cpr.2008.10.003
